# Multi-echo, multi-slice, cardiovascular T2* spiral imaging in a single breath-hold

**DOI:** 10.1186/1532-429X-18-S1-W31

**Published:** 2016-01-27

**Authors:** Nii O Addy, R Reeve Ingle, Kenneth O Johnson, Galen D Reed, Michelle M Nystrom, William R Overall, Juan M Santos, Bob S Hu

**Affiliations:** grid.420669.fHeartVista, Inc, Menlo Park, CA USA

## Background

Elevated levels of iron in the body can be detected in various organs including the heart. If not treated, high iron levels can lead to serious health conditions including heart failure and cirrhosis. Cardiovascular MR provides a non-invasive and repeatable alternative method to endomyocardial biopsy for monitoring iron levels within the myocardium.

Multi-echo, single slice, Cartesian acquisitions have previously been developed for cardiovascular iron assessment with MR, but the presence of iron within the myocardium can be heterogeneous [1]. In this work, we present a single breath-hold, multi-echo, multi-slice, spiral acquisition providing large coverage of the heart for the assessment of cardiovascular iron deposition.

## Methods

**Figure 1:**
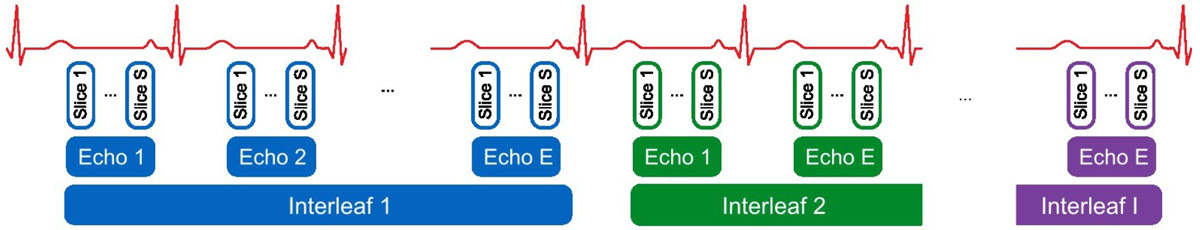
**Pulse sequence diagram of the multi-echo, multi-slice imaging sequence acquiring S slices and E echoes using a spiral trajectory with I interleaves**.

Imaging was performed on a 1.5 T GE Signa TwinSpeed scanner using the RTHawk Cardiac platform (HeartVista, Menlo Park, CA). A generalized pulse sequence diagram is shown in Figure 1 for S slices, E echoes and a spiral trajectory with I interleaves. Data was acquired with an ECG-gated GRE sequence with parameters: 28 × 28 cm^2^ FOV, 1.8 × 1.8 mm^2^ spatial resolution, four TEs (3.42 ms, 8.95 ms, 14.5 ms, 20.0 ms), 10 mm slice thickness, 4 spiral interleaves, 14.4 ms readout duration, and 1.2 acceleration factor. The desired number of slices to be acquired was determined by the user. Images were obtained within a single breath-hold by interleaving acquisitions from multiple slices within each heartbeat. Adjusting the number of slices affected the acquisition window within each heartbeat, but did not affect the overall scan time.

The sequence was tested on volunteers to demonstrate the feasibility of the technique. Images were reconstructed with partially parallel imaging with localized sensitivities.

## Results

**Figure 2:**
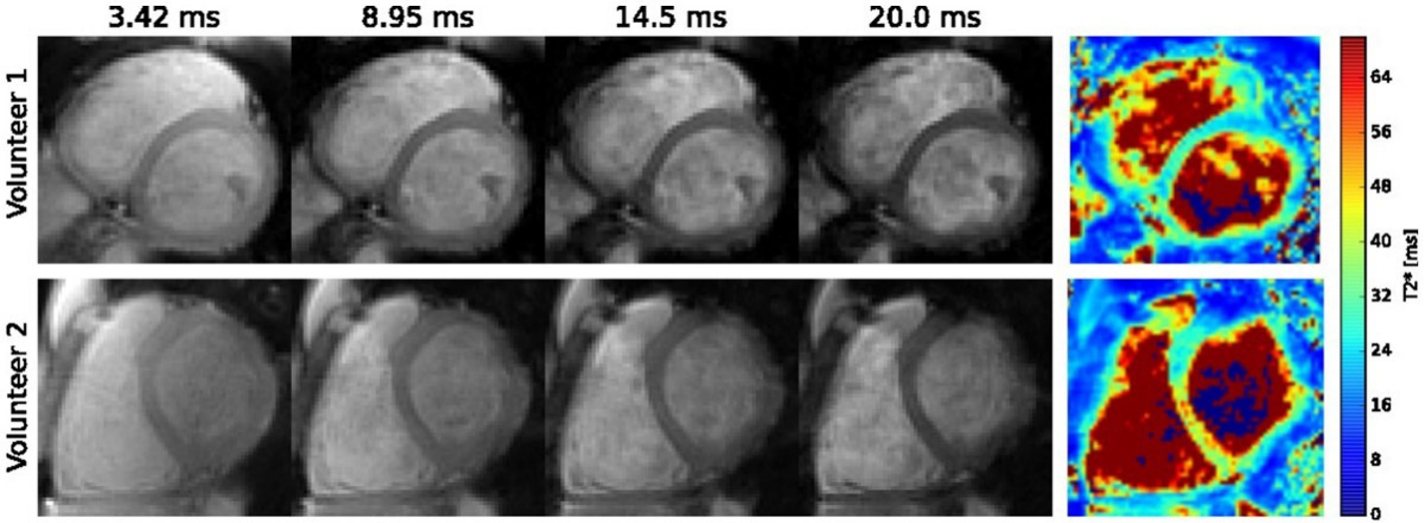
Magnitude images and T2* map based on the set of multi-echo images with TEs of 3.42, 8.95, 14.5, and 20.0 ms, for volunteers 1 (top) and 2 (bottom).

In volunteers 1 and 2, data was acquired in 16 and 20 s breath-holds, respectively. A single slice was imaged in volunteer 1 and 5 slices were imaged in volunteer 2. The images of the set of four TEs are shown in Figure 2. The signal intensity of the magnitude images were fit to a monoexponential curve M_0_e^-TE/T2*^ on a pixel-by-pixel basis to generate the T2* maps. T2* was measured in the interventricular septum, which is less prone to susceptibility artifacts and representative of T2* throughout the myocardium. The average T2* values in the two volunteers were 25.3 and 36.2 ms with standard deviations of 3.3 and 8.5 ms, respectively. Values greater than 20 ms are generally considered normal.

## Conclusions

With spiral imaging, a set of T2* weighted images can be acquired over multiple slices in a single breath-hold. Future work includes clinical evaluation.

